# Health Research Profile to assess the capacity of low and middle income countries for equity-oriented research

**DOI:** 10.1186/1471-2458-6-151

**Published:** 2006-06-12

**Authors:** P Tugwell, C Sitthi-Amorn, J Hatcher-Roberts, V Neufeld, P Makara, F Munoz, P Czerny, V Robinson, Y Nuyens, D Okello

**Affiliations:** 1 Centre for Global Health, Institute for Population Health, University of Ottawa, Ottawa, Canada; World Health Organization Collaborating Centre for Health Technology Assessment, Ottawa, Canada; Ottawa Hospital Research Institute, Ottawa, Canada; 2 Institute of Health Research, Chulalongkorn University, Bangkok, Thailand; 3 World Health Organization Collaborating Centre for Health Technology Assessment, Ottawa, Canada; 4 McMaster University, Hamilton, Canada; 5 World Health Organization; 6 Latin American Centre for Health Systems Research (CLAISS); 7 Coordinator, Health Research Profile Project (at time of Project); 8 Centre for Global Health, Institute for Population Health, University of Ottawa, Ottawa, Canada; 9 Coordinator, Council on Health Research for Development (at time of project); 10 World Health Organization, Mbabane, Swaziland; 11 Industry Canada, Broadband Program Office, Canada; 12 Free-lance consultant

## Abstract

**Background:**

The Commission on Health Research for Development concluded that "for the most vulnerable people, the benefits of research offer a potential for change that has gone largely untapped." This project was designed to assess low and middle income country capacity and commitment for equity-oriented research.

**Methods:**

A multi-disciplinary team with coordinators from each of four regions (Asia, Latin America, Africa and Central and Eastern Europe) developed a questionnaire through consensus meetings using a mini-Delphi technique. Indicators were selected based on their quality, validity, comprehensiveness, feasibility and relevance to equity. Indicators represented five categories that form the Health Research Profile (HRP): 1) Research priorities; 2) Resources (amount spent on research); 3) Production of knowledge (capacity); 4) Packaging of knowledge and 5) Evidence of research impact on policy and equity. We surveyed three countries from each region.

**Results:**

Most countries reported explicit national health research priorities. Of these, half included specific research priorities to address inequities in health. Data on financing were lacking for most countries due to inadequate centralized collection of this information. The five main components of HRP showed a gradient where countries scoring lower on the Human Development Index (HDI) had a lower capacity to conduct research to meet local health research needs. Packaging such as peer-reviewed journals and policy forums were reported by two thirds of the countries. Seven out of 12 countries demonstrated impact of health research on policies and reported engagement of stakeholders in this process.

**Conclusion:**

Only one out of 12 countries indicated there was research on all fronts of the equity debate. Knowledge sharing and management is needed to strengthen within-country capacity for research and implementation to reduce inequities in health. We recommend that all countries (and external agencies) should invest more in building a certain minimum level of national capacity for equity-oriented research.

## Background

Today, globalization threatens the health of our society with an undesirable effect on equity in health for development. The notion that Essential National Health Research (ENHR) is a key strategy for equity in development within and between countries is being revisited under the call for National Health Research Systems[1]. Indeed, the 2004 Mexico Ministerial Summit on Health Research concluded that: "*All countries, including the least developed, need the capacity to conduct health research, to implement and evaluate policies and programmes, and to communicate and use what is learnt*."[2].

This paper describes the results of an international survey, funded by the Council on Health Research for Development (COHRED), conducted in 12 low and middle income countries to develop a framework to assess the strength of national health research systems to improve population health and health equity.

The 1990 Commission on Health Research for Development stated that, "*for the most vulnerable people, the benefits of health research offered a potential for change that has gone largely untapped*"[3]. The primary recommendation from this report was that "...*each developing country should build its research capacity and conduct Essential National Health Research*." In the area of financing, the report recommended that developing countries invest at least 2% of their national health expenditures in research, and donors should invest at least 5% of their health budget in research and capacity building.

The 1990 Commission report led to the establishment of the Council on Health Research for Development (COHRED) with the mission to promote, facilitate, support and evaluate the role of ENHR strategies in strengthening health research systems, with an emphasis on health equity.

*"Equity in health implies that ideally everyone should have a fair opportunity to attain their full health potential and, more pragmatically, that no one should be disadvantaged from achieving this potential, if it can be avoided*"[4]. This definition requires a normative judgment of fairness and is therefore difficult to measure. A more recent definition of health equity that avoids this normative judgment is: "*Health disparities/inequalities are potentially avoidable differences in health (or in health risks that policy can influence) between groups of people who are more and less advantaged socially; these differences systematically place socially disadvantaged groups at further disadvantage on health" *[5] For example, child mortality is 2–5 times higher in the poorest compared to the richest in developing countries and has been described a health inequity that can and should be addressed by improved health systems [6].

In 1996, the World Health Organization Ad Hoc Committee for Health Research recommended the development of a special programme for research and training on health policy and systems[7]. Furthermore, the Ad Hoc Committee recommended the development of national research agendas using priority-setting with involvement of all relevant stakeholders (including policy-makers, researcher institutions, private sector, health care providers).

The Global Forum for Health Research was created in response to the report of the 1996 WHO Ad Hoc Committee. The Global Forum is an international forum for stakeholders to review global health research priorities, promote ongoing analysis of the international health research situation and facilitate coalition building to help correct the 10/90 gap, i.e. only 10% of global health research funds address 90% of the world's health problems [8].

In October 2000, the Bangkok conference on Health Research for Development [9] reviewed the extent to which the recommendations of the 1990 Commission had been implemented. The Bangkok Action Plan described three essential components of a national health research system as:

• Coherent and coordinated health research strategies and actions that are based on mutually beneficial partnerships between and within countries;

• An effective governance system; and

• A revitalized effort from all involved in health research to generate new knowledge related to the problems of the world's disadvantaged, and to increase the use of high quality, relevant evidence in decision-making.

## Methods

### Study design

We used both quantitative and qualitative methods to develop a framework for assessing the capacity of low and middle income countries' health research systems to address health equity issues and improve population health. We pilot-tested this framework in 12 low and middle income countries, using key informants to gather existing data from countries using a common conceptual framework (see below). Because the quantitative data from developing countries is of variable and low quality, we relied on the key informants to identify relevant data sources in each country and to reflect on the meaning of the data collected. These key informants were selected from within the mainstreams of health research systems, either from the academic or the government sides.

We choose three countries in each of four regions with different HDI levels and then compared trends across HDI levels within each region. This study provides a basis for generating hypotheses on the relationship of HDI to national health research systems.

### Developing the conceptual framework

**Figure 1:**
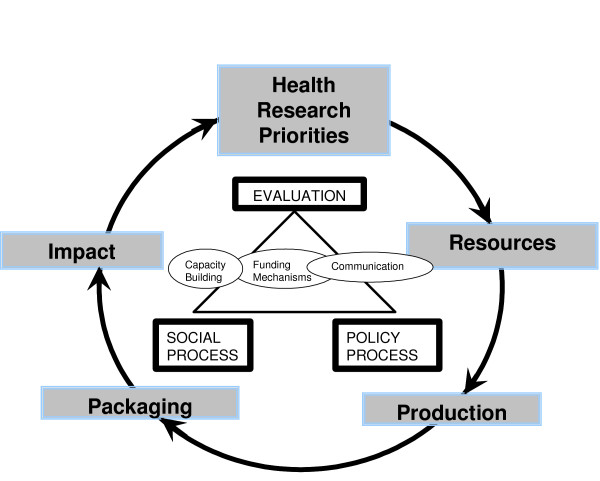
Health Research Profile Conceptual Framework: Health Research System Assessment Iterative Loop.

We developed a conceptual framework (figure 1) that recognizes that an idealized health system is highly adaptable and evolvable, with health research that functions to protect the health system from the damage of changing contexts the world is facing, such as globalization, privatization, global warming and threats from terrorism. In this conceptual framework, the health research system is characterized by five linked components: health research priorities, resources (amount spent on research), production (capacity issues), packaging, and impact (evidence of research affecting policy development or programs and interventions). Central to this framework is the "triangle that moves the mountain" [10].

The goal of this conceptual framework is to suggest processes for managing knowledge, making knowledge accessible to all stakeholders, to interpret the results within the political and social context and to facilitate decisions to improve population health and health equity.

We selected indicators for each component, using the principles described below.

Health Research Priorities refers to an analysis of knowledge gaps, fragmentation, and redundancies that ultimately produce the most cost-effective investment in knowledge production that is relevant to the local context.

Resources refers to financial resources as well as human and institutional capacities (eg number of researchers per capita), infrastructure, and research environment necessary to sustain an effective health research system. Human capacities include not only the supply of knowledge, but also the demand for knowledge to enhance equity in health for development.

Production refers to the capacity of the research systems to produce relevant output for policy-making such as whether research is produced in time to be useful to policy-making.

Packaging refers to the synthesis of knowledge in appropriate language and formats for different intended audiences (e.g. publications for researchers, lay summaries for policy-makers in governments, and research forums and networks for civil society) involved in policy and social processes leading to optimal health action and health equity. Packaging is essential to encourage the uptake and translation of research into improved health of the population.

Impact refers to evidence that knowledge from the research is used; i.e. debated by stakeholders with differing values then incorporated into policies accepted by the intended beneficiaries.

### Developing the indicators

The planning committee designed 14 catalytic questions about national health research systems, based on the conceptual framework, to assess whether countries had accepted the importance of priority setting at the national level, and the extent to which their responses consider health equity (questions available from the corresponding author). The four regional coordinators (CSA, PM, FM and DO) conducted a preliminary assessment of data availability for these questions in their regions (Asia, Central and Eastern Europe, Latin America and Africa) prior to a project meeting to select indicators for the HRP framework.

We then held a face-to-face project meeting in Geneva (Oct 21–22, 1999) to brainstorm indicators using a "mini-Delphi" process [11,12]. A panel of experts (HRP team members) first identified the high-level issues to be addressed in the HRP and the criteria and principles for selection of indicators. Then the Delphi technique was applied as follows:

**Step 1.**The experts were asked to nominate any indicators for national health research they thought would be useful to the study – 40 indicators were compiled. Most of these indicators were derived from other initiatives such as the Global Equity Gauge Alliance, the UNDP Human Development Report, the ENHR indicators, OECD indicators and the ASEAN Multi-country study on resource flows for health research and development.

**Figure 2:**
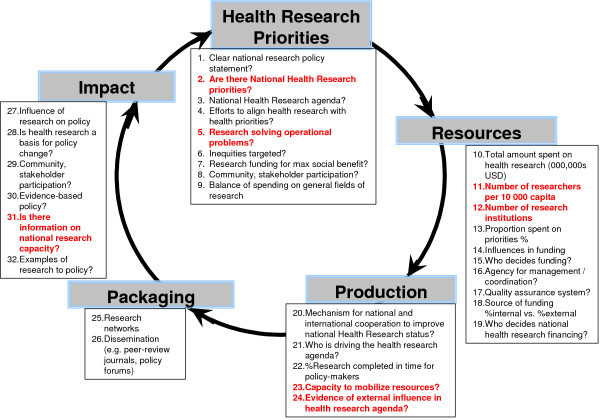
HRP Framework and indicators. Indicators in **bold red **could not be measured by >33% of countries.

**Step 2.**The participants were asked to select their five most preferred indicators based on quality, validity, comprehensiveness, feasibility and relevance to equity. Clusters of choices for the initial list of indicators were noted by the facilitator and consolidated into five indicator groups comprising 32 indicators shown in Figure 2.

### Selecting the countries

The project team, consisting of a regional coordinator from each of the continental regions of Africa, Asia, Latin America and Central and Eastern Europe, selected three countries from each region to represent low, middle and high scores on the Human Development Index (HDI) [13]. The HDI measures a country's achievements in terms of life expectancy, educational attainment and adjusted real income. We hypothesized that countries with a higher HDI might have stronger health research systems. In addition, we ensured that the selected countries had a range of experience in implementing the Essential National Health Research Strategy.

We selected Korea, Thailand and Bangladesh in Asia; Hungary, Lithuania and Kazakhstan in Central and Eastern Europe; Mauritius, Namibia and Uganda in Africa; and Chile, Ecuador and Nicaragua in Latin America (Table 1).

In each of these countries, the regional coordinator worked with a country collaborator who held consultations with researchers, research managers and representatives of government and non-government organizations to determine the feasibility of obtaining information on the HRP indicators. The regional coordinators and country collaborators were responsible for seeking opinion leaders from all relevant stakeholders in the health research systems from each country.

The regional coordinator and country collaborator obtained information on the 32 HRP indicators from both documents and discussions with stakeholders in each country in 2000. Where the direct indicators were not available, proxy indicators were selected which the advisory working group felt best represented the situation.

### Feasibility of data collection

Most indicators (26 out of 32) were answered by at least 8 countries (67%). Despite the lack of data for the other 6 indicators, these indicators provide important information that we need to develop measurement systems for these components of a country's national health research system. The other indicators that were less feasible to measure involved interpretation of available data, such as the capacity to mobilize resources and whether research is solving operational problems. However, we were surprised at the lack of information on the number of researchers and research institutions in each country.

Some of the indicators involved subjective assessments, such as whether research funding is allocated for maximum social benefit and the degree to which research is available "on-time" for policy makers. The project team ensured a common understanding of these subjective assessments with the country representatives as well as the country respondents.

## Results

### Health research priorities (Table 2)

Implicit in setting research priorities is the analysis of knowledge needs and knowledge available for health decisions and actions. Six out of 12 countries reported having both a national health research agenda of some kind and efforts to align research with priorities. Three countries reported research agendas showed alignment with wider health priorities. Two countries had neither a research agenda nor alignment (Chile, Korea), and one country reported a research agenda but no alignment (Kazakstan).

In Chile, although there is no clear policy statement, the Ministry of Health established a Commission for Research and Technology whose purpose is to support the Minister in promoting research projects directed toward high priority health issues. This effort is convening a working roundtable of representatives from the Faculties of Medicine who will consider the basis of an ENHR policy. A first step in this direction is represented by the establishment of a small fund of US$1.5 million addressed specifically to research on national health priorities. The fund is managed both by the Ministry of Health and the National Council of Science and Technology. Similarly, in Lithuania, while there is no list of national health research priorities, there is a clearly stated policy on the "National Concept of Health" and the Ministry of Health has prioritized four areas for its Research Support Fund. In another case with no explicit priorities, Nicaragua, a review of 59 projects showed an important link between research and national health priorities as judged externally, likely due to a concordance between interests of funding agencies and the reality of a country in the lower level of human development.

Where national priorities or agenda were identified, strategies included the implementation of the ENHR strategy (Uganda, Namibia), other national government policy statements (Lithuania), a government task force (Ecuador) and ministry of health efforts (Hungary).

Some barriers to implementation included changes in government (Ecuador), lack of support from the prime minister's office or government for ministry of health efforts (Hungary). In some countries, researchers and research institutes can access funds for health research which does not meet the national priorities (eg Thailand).

### Targeting inequities

Half of the respondents indicated specific research programs to address inequities in access to services and health status across the socioeconomic factors of gender, urban-rural differences and income (**Indicators 6 & 7**). Three countries described no research addressing inequities or social benefit (Korea, Kazakhstan, and Ecuador).

In three countries, there was significant research on inequities (Chile, Mauritius, Namibia, Nicaragua). In Chile, the Chilean Equity Gauge, led by Jeanette Vega, defined national equity objectives [14]. In Mauritius, the national health sector reform considered health equity in developing the Health Insurance System which provided health care for every Mauritian, irrespective of ability to pay. Mauritius also has conducted studies of health status inequities. In Namibia, the District Health Survey (DHS), documented accessibility of adequate housing, water, sanitation, specific health services, including facilities as well as inequities in health status relative to socio-economic conditions, literacy and age. The Namibia DHS now includes a wealth index which stratifies respondents into quintiles to enable further important analysis on inequity. In Nicaragua, the driving force in inequity research was external funding related to gender issues.

In other countries, equity research was insufficient, according to country collaborators. For example, in Hungary, the Ministry of Health has conducted some studies of geographical access to health services. In Thailand, the research addressing the issues of inequity has been non-systematic and cannot keep pace with the trend in globalization. There is, however, capacity and data available to measure the gap across socioeconomic indicators for some indicators of health status and access. In Uganda, adoption of ENHR and research debates of key stakeholders raised awareness about accessibility issues that are enshrined in a new health services policy plan aimed to deliver health to the rural poor. However, research on inequities was considered insufficient.

Barriers to inequity research described by country respondents included the lack of measurement and monitoring data to assess the gap between socioeconomic groups (Bangladesh, Ecuador), small research studies with little ability to impact policy (Korea), little research on social determinants of health (Namibia) and little real national political commitment (despite verbal commitment) (Lithuania).

### Balance of spending on general fields of health research

In six countries, the majority of research was bio-medical and clinical from 80% in Hungary to 46% in Thailand **(Indicator 9)**. Systems research was the next highest ranked field of research in five of seven cases, most notably in Namibia where it received 98% of funding. Only Nicaragua reported significant research on public health (51% of funds), followed by biomedical (27%) and systems research (22%).

### Resources (Table 3)

Four countries (Mauritius, Namibia, Uganda and Nicaragua) were unable to report the amount of health research financing or the number of researchers or research institutes **(Indicators 10, 11, 12 and 13).**

The most common problem was that there did not seem to be any centralized data collection, and a high degree of fragmentation in the research system so data was available for some research projects, but not the country as a whole. In some cases, this was related to issues of institutional secrecy. In other cases, high degrees of external funding made national estimates difficult **(Indicator 18).**

For those countries which reported a total amount spent on health research (**Indicator 10**), there was a clear gradient according to HDI, with the highest amount of funding in the highest HDI country of each region. Furthermore, the higher the HDI, the lower the proportion of foreign or international sources of funding ranging from 6% external funding in Hungary, 97% in Namibia and 99% in Uganda (**Indicator 18)**. Furthermore, there is evidence of a high degree of foreign influence on funding decisions in 6 out of 12 countries(Bangladesh, Mauritius, Namibia, Uganda, Ecuador and Nicaragua) (**Indicator 19**). In two high HDI countries (Mauritius and Chile), researchers have a significant role in deciding research funding.

In terms of human resources, **(Indicator 12) **where data were available, following the trend of finances, the proportion of researchers in a country decreased along with a score on the HDI. For example, in the CEE countries, Hungary had 4.2 researchers per 10 000 population, Lithuania had 3.6, and Kazakhstan 2.1.

The count of research institutions was only answered by 6 countries, with the average number of institutes of 15 **(Indicator 13)**. However, this count of institutes can be confused by a count of large institutions that contain identifiable research entities such as a ministry or university versus actual institutes or research sites, some of which may be quite small by comparison.

#### Quality assurance and national coordination

All countries except Korea, Mauritius and Nicaragua reported some type of coordination system for health research.

Of the nine countries with a quality assurance system, the systems included mandatory rules for publishing in international journals (Chile), a national research council that must approve any research project before it is funded (Korea, Kazakhstan, Mauritius, Thailand, Namibia) and professional peer review (Hungary, Chile, Lithuania, Nicaragua).

### Production (Table 4)

We used 4 indicators to assess national production capacity (indicators 14,19,23 and 24- influences in research funding, who decides financing, capacity to mobilize resources and external vs internal source of funding).

The national research agenda was described as driven by researchers (6 countries), foreign aid, the prime minister and ENHR. There were three countries which fund and direct their own agendas (Hungary, Lithuania, Thailand- with some foreign funding). Four countries showed a high degree of foreign/multinational influence (Bangladesh, Namibia, Ecuador and Nicaragua). In two cases, there was a high degree of foreign funding with a domestic capacity to direct the funds (Chile, Mauritius). Three cases did not report enough information to assess both funding and capacity (Uganda, Kazakhstan, Korea).

Whether research was completed on time for policy-makers was answered by only one country (Hungary- 39%).

Barriers in production capacity cited by respondents included fragmentation of research (e.g. Bangladesh, Hungary, Lithuania, Mauritius), pharmaceutical research unrelated to country priorities (Chile), researcher-driven agendas leading to duplication (Ecuador, Hungary), small projects (e.g. in Thailand, 65% of projects were small) and a low social status and poor promise of career track for researchers (reported by 7 out of 12 countries).

In Namibia, there is a healthy discourse and competitive engagement around the determination of the research agenda, which includes players such as policy makers, MOHSS program managers, researchers, donors and civil society which contributes to greater focus on reducing gaps in knowledge. In other countries, emerging efforts to reduce fragmentation are the emergence of an overall National Research and Development body to reduce duplication and fragmentation in Chile and the increasing involvement of civil society in demanding equity-oriented research in Korea.

In Thailand, there is an effort to address issues of fragmentation of the research system by improving governance of the health research system. Good governance includes mobilization of financial resource according to priorities, strengthening capacities for research, research management, good quality research products and the appropriate use of knowledge for debates by the various target groups (citizens, NGOs, governments) in their approach to conflict resolution.

### Packaging (Table 5)

Most countries (9 out of 12) answered affirmative to having some form of dissemination, either using research networks or peer-reviewed publications **(Indicators 25 & 26)**.

Countries reported varying abilities to describe the number of peer-reviewed publications. For example, Nicaragua reported that >90% of research projects are published, Thailand reported 38% of work published whereas in Ecuador, a review of 30 projects revealed that only 3 (10%) were published in peer-reviewed journals. We found a trend in number of publications by country, according to the HDI, using an electronic search in Medline in 1999 (Table 6). This trend did not apply to Africa, likely due to the strong research infrastructure at Makerere University in Uganda which had close ties with the University College of London in the UK. Furthermore, the higher HDI countries chosen in Africa are small (Mauritius 1.2 million and Namibia 2 million in 2005).

In Namibia, research results are increasingly presented at formal fora, which in itself introduces a measure of peer review, and strengthens networking, cross-fertilization and quality enhancement.

Barriers to dissemination described by respondents were research that is not completed (e.g. 55% of research projects in Thailand were not completed) and low quality research (Mauritius).

Dissemination to non-scientific audiences was described by Thailand and Mauritius. Thailand produces videos for the general public as well as policy-maker briefs for some projects [15,16]. In Mauritius, research is packaged for dissemination to the public through the media and workshops.

### Impact (Table 7)

Eleven of the twelve countries in the study offered examples of research whose result has influenced policy **(Indicators 27, 28, & 32). **However, seven of these countries qualified the influence on policy as rare or limited. Most countries were able to give specific examples of projects which had influenced policy. Nicaragua reviewed 59 projects, and determined that 22 of these projects could be linked to decision-making at a policy level. In Thailand, projects on infectious disease had not only influenced policy, but had also sustained core funding to continue their work. One country reported that the impact of research on policy was worse for poor people and disadvantaged areas (Korea).

Mechanisms for influencing policy included national fora (Thailand) and government commissioned research (Chile, Hungary). In Thailand, a health forum allowed stakeholders in the policy and social process to revitalize health systems, re-strategize research and service institutions, build in accountability for actions, and harness allocative efficiency **(Indicator 29)**. In Chile, the ministry of health commissioned research on malnutrition which led to changes in food supplementation policies. In Hungary, health care system research has a direct mutual link to political decision making, through the Health Development Research Institute. Results are used as preparatory material for decision making.

Some barriers to influencing policy include frequent changes in government (Ecuador), a communication problem between researchers and decision-makers (Hungary), small studies that do not generalize to the whole country (Mauritius) and the difficulty to strike an appropriate balance between fulfilling the curiosity of the supply of health research (researchers and research establishment) and the demand of health systems to promote equity.

## Discussion

This study developed a common conceptual framework to collect data on national health research systems, using a combined quantitative and qualitative approach that used existing data from countries, supplemented by interpretation by key informants from within the health research systems in countries. We then pilot-tested this framework in 12 low and middle income countries. Our findings of consistent trends across HDI in the different regions for a range of indicators provide the basis for construct validity that our framework can be used to generate hypotheses and design future studies to address weaknesses of using existing datasets.

In our conceptual framework, unmet health needs and societal values, including equity, are the foundation of the health research system. This framework and the indicators selected argue that a social and political process of all stakeholders is required to effectively address health problems, while maintaining the underlying values of society. We propose that this framework may be used to activate the political process and social process to make the health research system more responsive to the needs of the health system particularly regarding its underlying value (e.g. equity as the underlying value – which might not be true for all countries).

Weakness of data from developing countries is a limitation of this framework which draws on existing datasets, which we addressed by drawing on the knowledge of key informants experienced in the country health research systems to interpret the data. This study selected only 12 countries which limits the external generalizability of the study.

A strength of our framework is that both the framework and indicators were developed with the full participation of our colleagues in decision-making and execution of activities in the selected regions and countries. Partnership with our colleagues also involved mentoring and capacity building throughout the project inception, development, execution and dissemination.

The World Report on Knowledge for Better Health is developing and testing a set of 43 indicators for the strength of health research systems based on their four part framework of stewardship, financing, resources and producing and using research [17]. The HRP framework includes several comparable indicators, but has a greater focus on measuring the impact and packaging of health research on health policy and population health outcomes. Furthermore, our framework and approach uses both academics and government officials to collect and interpret data from countries. Comparison of results of our framework and the WHO framework may also lead to generation of hypotheses regarding how to measure these indicators and their relevance for priority-setting at the national level.

We identified five research issues based on the results of this project: equity, knowledge management, research priorities, funds and funding, and evaluation.

### Equity

The need to clarify the extent of health inequities in health research systems amongst countries is clearly of concern, since only 7 out of 12 countries stated that inequities were targeted as part of health research priorities (Table 2). Furthermore, those countries with the lowest HDI, tended to score the lowest, indicating a between country inequity in the ability of national health research to address local health problems and needs.

We need research on whether health reform efforts being explored in most countries will result in improved health equity. For example, improvement in average indicators such as childhood mortality has been shown to obscure stagnant or worsening gaps between income quintiles [6].

### Knowledge management

Knowledge management refers to the packaging and implementation of research results so that the research results are available and used to make decisions about health policies and programs. Our results show that research is packaged for scientific publications and research networks. However, the extent to which knowledge is packaged for other audiences is unclear from our data. Furthermore, problems of low quality and ability to complete research limit the ability to disseminate knowledge.

We need to evaluate mechanisms to increase knowledge management and knowledge translation and their impact on health equity. For example, what is the impact on population health and health equity of national research fora described in Thailand and Africa that engage diverse stakeholders including intended beneficiaries, authorities with formal power, private sectors and civil society [18,19] How should these mechanisms consider cultural and societal values? For example, members of the International Clinical Epidemiology Network (INCLEN) recently found that physicians are more likely to adopt practices if research has been carried out locally [20].

### Research priorities

Research priorities can be used to review resources, knowledge production, knowledge packaging, and measurements of impact to determine whether health research needs have been met. Only 3 out of 12 countries reported national health research priorities, and several countries were not able to answer indicators related to whether health research is meeting needs and whether health research is having an impact. Since 1999, various pragmatic approaches for priority setting have been developed including the Combined Approach Matrix (CAM), which advocates for a transparent, iterative, equity-oriented, multidisciplinary approach involving all relevant stakeholders [21]. Our findings indicate that research is needed on how to facilitate priority setting at the national level in low and middle income countries.

### Funds and funding mechanisms

The Commission on Macroeconomics and Health concluded that increased investment in health research by both countries and donors is needed to realize gains in social and economic well-being that are essential to meet the Millennium Development Goals. We found a large degree of foreign external funding, as well as external influence on the health research agenda of many countries.

To more effectively strengthen knowledge systems, we propose that a stable source of funds is required from national governments. Since 2000, there has been considerable progress towards meeting the 1990 Commission goal of 2% of health budgets towards research in low and middle income countries. However, only two countries (Brazil and Cuba) were close to 2%, and in all countries, health spending represents only a small fraction of the GDP of the country.

Research is needed to assess whether external investments in research and capacity strengthening are in line with national priorities and needs. Furthermore, research is needed on successful transitions from dependence on external funding to greater within-country funding and sustainability.

### Impact and evaluation

We found poor ability to measure the impact of research on population health and health equity. We identified some barriers to influencing policy including frequent changes in government, communication problems between researchers and decision-makers, small studies that do not generalize to the whole country and the difficulty to strike an appropriate balance between fulfilling the curiosity of researchers and the demand of health systems to promote equity.

More consistent and systematic evaluation is required to assess the impact of national health research systems on population health and health equity. The Health Metrics Network launched in 2004 by the World Health Organization may contribute to improved availability of longitudinal data on equity in health [22]. For example, are regional health research and development fora, such as the Africa Forum [23] and the Asia Health Research Forum [24] that are based on inclusiveness, country-focus and ownership, succeeding in improving equity in development through research in health?

## Conclusion

It is evident that the efforts of COHRED and other agencies have succeeded in bringing research on the national health development agendas, for example, by the Mexico Ministerial Statement on health research. However, countries in the lower HDI group are still a long way from being able to translate the research agenda into operational programmes. In particular, nearly all the countries do not have clear national research priorities; and there is limited use of research to solve operational problems, address country priorities (particularly equity issues) or influence policy.

In countries with a higher HDI, there was evidence of greater capacity (human resources, research institutions, publications, financial resources) but no greater link to equity-focused research, alignment of research with health priorities or use of research for policy-making. Although we conducted no formal statistical tests for trend, the consistent trend across HDI provides evidence of construct validity for the common framework. Hence, the results provide the basis for generating hypotheses about the relationship between human development indicators and national health research investment.

Our data indicates that the research agendas in some countries may still be driven by institutions created by COHRED. Without a strong institutional framework and a clear national budget support line, it will be difficult to operationalise the well articulated concepts from the 1990 Commission, and the impact of research will not be easy to define.

There is clearly an indication that research may not yet be fully integrated in the operations of health programmes. This integration of health programmes and research is something countries should strive to do, and to avoid the "project mentality" on matters of research.

Another area of concern is that the capacity for research (human, research institutions, total funding, etc) is very low in nearly all the countries. This obviously has a direct linkage with the production level, which is also low. It is therefore not surprising that there is no link between research done and ability to solve operational problems.

We found that while there is indeed research on health determinants such as socioeconomic status, education and gender and its relationship to health and well being, research on the impact of those determinants on issues such as access to the health care system was very limited. We need to reflect on the fact that equity issues were consistently under-represented in setting research priorities, conducting research projects and ability to influence policy decisions.

In conclusion, we recommend that all countries (and external agencies) should invest more in building a certain minimum level of capacity for research in the countries so we can reap the benefit of the recommendations of the 1990 Commission on Health Research. We need to evaluate the impact of new and ongoing initiatives to bridge the "Know-Do" gap between research and action and improve the translation of research into improved health and health equity [25]. These knowledge translation activities are essential for the achievement of the Millennium Development Goals.

## Competing interests

The author(s) declare that they have no competing interests.

## Authors' contributions

PT, YN, CS and JHR conceived the idea for this study and participated in the design and coordination. PM, FM, DO, CS led regional teams. PC and VR coordinated the collection of data and synthesis of results and helped draft the manuscript. All authors read and approved the final manuscript.

## Pre-publication history

The pre-publication history for this paper can be accessed here:



## Figures and Tables

**Table 1:** Countries surveyed and their Human development index (HDI)

**Region**	**Low**	**Medium**	**High**
**Africa**	Uganda HDI = 0.34	Namibia HDI = 0.507	Mauritius 0.833
**Central and Eastern Europe**	Kazakhstan 0.74	Lithuania HDI = 0.761	Hungary HDI = 0.795
**Asia**	Bangladesh HDI = 0.44	Thailand HDI = 0.753	Korea 0.875
**South America**	Nicaragua HDI = 0.635	Ecuador HDI = 0.767	Chile 0.893

**Table 2:** Health Research Priorities

**Function Elements & Related indicators**	**Asia**			**CEE**			**Africa**			**Latin America**		
	
	Korea	Thai	B'desh	Hung	Lithu.	Kazak.	Mauri.	Namib	Uganda	Chile	Ecuad	Nicar.
1. Clear national research policy statement?	N	Y	N	N	Y	N	N	Y	Y	N	Y	N/A
2. Are there National Health Research priorities?	N/A	Y	Y	Y	N	N/A	N/A	N/A	N/A	N	N/A	N/A
3. National Health Research agenda?	N/A	Y	Y	Y	Y	Y	Y	Y	N	N	N	N
4. Efforts to align health research with health priorities?	N	Y	Y	Y	Y	N	Y	Y	Y	N	Y	Y
5. Research solving operational problems?	N	N/A	N/A	N	Y	N	N/A	N/A	N/A	N/A	N/A	N/A
6. Inequities targeted?	N/A	Y	Y	Y	Y	N	Y	Y	N	Y	N	N
7. Research funding for maximum social benefit?	N	Y	Y	Y	Y	N	N	Y	Y	N	N	N
8. Community, stakeholder participation?	N/A	N	Y	N	N	N	Y	Y	N/A	Y	N	N
9. Balance of spending on general fields of research.	N/A Beh 28%	Bio 46% Sys 20% Beh 2%	Bio 13% Sys 14% Beh 3%	Sys 17% Bio 80%	Bio 75% Sys 5% Beh 20%	Bio majority	N/A Beh 2%	Sys 98% N/A	Beh 14% Bio 85%	Sys 1% Bio 30%	Sys 13% N/A	Public Health 51%

CEE: Central and Eastern  Europe; Thai: Thailand, B'desh: Bangladesh, Hung: Hungary, Lithu: Lithuania, Kazak: Kazakhstan, Mauri: Mauritius, Namib: Namibia, Ecuad: Ecuador, Nicar: Nicaragua, Beh: behavioural; Bio: biomedical; Sys: Health systems, Rs: Researchers, PMs: prime minister, ENHR: Essential national health research

**Table 3:** Resources

**Function Elements & Related indicators**	**Asia**			**CEE**			**Africa**			**Latin America**		
	
	Korea	Thai	B'desh	Hung	Lithu.	Kazak.	Mauri.	Namib	Uganda	Chile	Ecuad	Nicar.
10. Total amount spent on health research (000,000s USD).	140.3	2.3	2.3	24.9	22.5	2.4	N/A	N/A	N/A	10.7	2.2	N/A
11. Proportion spent on priorities. %.	N/A	N/A	N/A	60%	N	LOW	N/A	N/A	N/A	N	N/A	N/A
12. Number of researchers per 10 000 capita	4.4	1.77	0.042	1.54	3.6	2.1	N/A	N/A	N/A	N/A	N/A	N/A
13. Number of research institutions	N/A	28	20	20	8	14	N/A	N/A	N/A	4	N/A	N/A
14. Influences in funding.	N/A	N/A	FORGN	GOV	N/A	Rs	FORGN	FORGN	N/A	GOV	FORGN	FORGN
15. Who decides funding?	GOV	GOV	GOV FORGN	GOV	GOV	GOV	Rs	FORGN	FORGN	Rs	FORGN	FORGN
16. Agency for management/coordination?	N/A	Y	Y	Y	Y	Y	N	Y	Y	Y	Y	N/A
17. Quality assurance system?	Fin-ancing	Fin-ancing	N	Peer-review	Peer-review	Fin-ancing	Fin-ancing	Fin-ancing	N	Peer-review	N/A	Peer-review
18. Source of funding %internal vs. %external.	N/A	INT 95 EXT 5	INT 34 EXT 66	INT 94 EXT 6	INT 90 EXT 10	N/A	INT 1 EXT 99	INT 3 EXT 97	N/A	< EXT	< EXT	< EXT
19. Who decides national health research financing?	GOV	GOV	GOV FORGN	GOV	GOV	GOV	Rs	FORGN	FORGN	Rs	FORGN	FORGN

CEE: Central and Eastern  Europe; Thai: Thailand, B'desh: Bangladesh, Hung: Hungary, Lithu: Lithuania, Kazak: Kazakhstan, Mauri: Mauritius, Namib: Namibia, Ecuad: Ecuador, Nicar: Nicaragua, GOV: Government, FORGN: foreign funding, PLCY: Policy, AGDA: Agenda, INT: Internal funding, EXT: external funding, Rs: researchers

**Table 4:** Production

**Function Elements & Related indicators**	**Asia**			**CEE**			**Africa**			**Latin America**		
	
	Korea	Thai	B'desh	Hung	Lithu.	Kazak.	Mauri.	Namib	Uganda	Chile	Ecuad	Nicar.
20. Mechanism for national and international cooperation to improve national Health Research status?	National researchagenda	N/A	N/A	Funder and Stake-holders	Coop-eration	Policy	N/A	Coop-eration	N/A	N/A	N/A	N/A
21. Who is driving the health research agenda?	Rs PMs	Combo	Foreign Aid	Rs	Rs	Rs	Rs	Combo	ENHR	Rs	Foreign aid	Foreign aid
22. %Research completed in time for policy-makers.	N/A	N/A	N/A	39%	N/A	N/A	N/A	N/A	N/A	N/A	N/A	N/A
23. Capacity to mobilize resources?	N/A	HIGH FORGN	HIGH FORGN	LOW	LOW	N/A	N/A	N/A	N/A	N/A	N/A	N/A
24. Evidence of external influence in health research agenda?	N/A	N/A	Y	N/A	N/A	N/A	N/A	Y	N/A	Y	Y	Y

CEE: Central and Eastern  Europe;  Thai: Thailand, B'desh: Bangladesh, Hung: Hungary, Lithu: Lithuania, Kazak: Kazakhstan, Mauri: Mauritius, Namib: Namibia, Ecuad: Ecuador, Nicar: Nicaragua, FORGN: foreign funding, Rs: Researchers, ENHR: Essential National Health Research, Combo: combination, PMs: prime ministers

**Table 5:** Packaging

**Function Elements & Related indicators**	**Asia**			**CEE**			**Africa**			**Latin America**		
	
	Korea	Thai	B'desh	Hung	Lithu.	Kazak.	Mauri.	Namib	Uganda	Chile	Ecuad	Nicar.
25. Research networks	N/A	Y	Y	Y	Y	Y	N	Y	Y	Y	Y	N/A
26. Dissemination (e.g. peer-review journals, policy forums)	N/A	Y	Y	Y	Y	Y	N	N/A	N	Y	Y	Y

CEE: Central and Eastern  Europe; Thai: Thailand, B'desh: Bangladesh, Hung: Hungary, Lithu: Lithuania, Kazak: Kazakhstan, Mauri: Mauritius, Namib: Namibia, Ecuad: Ecuador, Nicar: Nicaragua

**Table 6:** Research publications with lead author from LMIC in Index Medicus

**Region**	**Low HDI**	**Middle HDI**	**High HDI**
**Africa**	Uganda 69	Namibia 2	Mauritius 2
**Central and Eastern Europe**	Kazakhstan 0	Lithuania 66	Hungary 950
**Asia**	Bangladesh 85	Thailand 735	Korea 2882
**South America**	Nicaragua 3	Ecuador 23	Chile 542

LMIC: low or middle income country, HDI: human development index

**Table 7:** Impact

**Function Elements & Related indicators**	**Asia**			**CEE**			**Africa**			**Latin America**		
	
	Korea	Thai	B'desh	Hung	Lithu.	Kazak.	Mauri.	Namib	Uganda	Chile	Ecuad	Nicar.
27. Influence of research on policy.	N/A	Y	Y	Y	Y	Y	N/A	N	Y	Y	N	N
28. Is health research a basis for policy change?	Y	Y	Y	N	Y	N	N	Y	N	N	N	Y
29. Community, stakeholder participation?	N/A	N	Y	N	N	N	Y	Y	N/A	Y	N	N
30. Evidence-based policy?	N/A	Y	Y	Y	Y	N	N/A	Y	N	Y	N	N
31. Is there information on national research capacity?	Y	Y	Y	Y	Y	Y	N/A	N/A	N/A	N/A	N/A	N/A
32. Examples of research to policy?	N/A	Y	Y	Y	Y	Y	N/A	N	Y	Y	N	N

CEE: Central and Eastern  Europe; Thai: Thailand, B'desh: Bangladesh, Hung: Hungary, Lithu: Lithuania, Kazak: Kazakhstan, Mauri: Mauritius, Namib: Namibia, Ecuad: Ecuador, Nicar: Nicaragua,
